# Construction and Immunogenicity of a Recombinant Pseudorabies Virus Variant With TK/gI/gE/11k/28k Deletion

**DOI:** 10.3389/fvets.2021.797611

**Published:** 2022-01-25

**Authors:** Shijun Yan, Baicheng Huang, Xiaofei Bai, Ying Zhou, Linghua Guo, Tongyan Wang, Yihong Shan, Yuzhou Wang, Feifei Tan, Kegong Tian

**Affiliations:** National Research Center for Veterinary Medicine, Luoyang, China

**Keywords:** pseudorabies virus (PRV), gene deletion, bacterial artificial chromosome, vaccine, immune protection

## Abstract

In China, the re-emerging pseudorabies virus (PRV) variant has caused large-scale outbreaks of pseudorabies in swine herds with classical PRV vaccine immunization since late 2011. Here, a recombinant PRV with TK/gI/gE/11k/28k deletion was constructed based on variant HN1201 strain isolated in 2012, by the bacterial artificial chromosome infectious clones. Compared with the parental virus, the recombinant PRV rHN1201^TK−/*gE*−/*gI*−/11*k*−/28*k*−^ showed a similar virus grown curve and exhibited smaller plaques. The vaccination of rHN1201^TK−/*gE*−/*gI*−/11*k*−/28*k*−^ could elicit an earlier and higher level of gB antibody, and the neutralizing antibodies elicited by rHN1201^TK−/*gE*−/*gI*−/11*k*−/28*k*−^ were effective against both PRV classical and variant strains. Clinically, the body temperature of the pigs immunized with rHN1201^TK−/*gE*−/*gI*−/11*k*−/28*k*−^ was significantly lower than that of the classical PRV vaccine immunized pigs, and the recombinant PRV could provide effective protection against the challenge with the PRV variant. These results imply that the rHN1201^TK−/*gE*−/*gI*−/11*k*−/28*k*−^ could be a promising vaccine candidate for the prevention of the current epidemic of pseudorabies in China.

## Introduction

Pseudorabies (PR), also called Aujeszky's disease, is caused by the infection of an alpha-herpesvirus Pseudorabies virus (PRV) (1). The double-stranded DNA genomic sequence of PRV is ~145 kb in size, containing almost 70 open reading frames (ORFs) that encode 70–100 viral proteins (2).

The herpesvirus PRV has a broad host range, which is known to cause acute fatal disease in a variety of mammals (3–5). The PRV infection may lead to acute symptoms and even death in piglets, and the clinical signs of coughing, sneezing, lethargy, nervousness, uncoordinated movements, and abortion in sows (1), resulting in heavy economic losses in the pig industry. Like other alpha-herpesviruses, PRV is characterized by a lifelong latent infection in the host peripheral nervous system. Stress-induced reactivation of latent PRV is a difficulty for PR prevention (6).

Highly efficacious gene-deleted modified-live vaccines, such as the strain PRV Bartha-K61 that attenuated from wild-type strain Bartha *via* multiple passages on pig kidney cells and chicken embryos, and their companion differential serological tests have been widely used to control PR during the past several decades (7, 8). Compared with the wild-type strain, the attenuated Bartha carries a large deletion in the unique short region of the genome, including the complete genes of gE and US9 (11k), and part of the US2 (28k) and gI (9). However, the emerging virulent PRV strains have caused severe PR in the vaccinated pigs in China since late 2011 (10–13), while the widely used PRV vaccines of classical strains only provide limited protection to the new-emerging PR (12). After the new-emerging PR outbreaks, a virulent PRV variant HN1201 in China was isolated, which induced high fever, anorexia, coughing, dyspnea, and systemic neurological symptoms in the infected pigs (12, 14, 15). Multiple studies have shown that the highly virulent PRV was the causal agent of this PR epidemic (12, 14–16). Therefore, it is urgent to develop more effective PRV vaccines based on the emerging PRV strains for the disease control.

Bacterial artificial chromosome (BAC) infectious clone is widely used for the studies of viral genome manipulation, and then be used for evaluating the efficacy of vaccine candidates (17–20). In herpesvirus, the BAC system was a powerful tool for generating recombinant viruses, which promotes the understanding of viral pathogenesis, vaccine development, and gene therapy (21). The gI, gE, and TK genes were critical for PRV virulence, but with no obvious effect on viral immunogenicity (22, 23). The gene of 11k is required for the efficient spread of PRV in the nervous system (24, 25). The deletion of 28k gene in the attenuated PRV vaccine strain strongly suggested an important role of 28k in virulence determination (26), and more recently, the 28k gene deletion showed an enhancement of PRV titers *in vitro* (27).

Here, a TK/gE/gI/11k/28k deleted PRV strain was generated based on a modified RPV, HN1201^TK−^ (15), using BAC infectious clone, and then the immunogenicity of the 5-gene-deleted vaccine candidate was evaluated in pigs.

## Materials and Methods

### Animals

Pigs (28-day-old) used in this study were tested free of PRV, porcine reproductive and respiratory syndrome virus (PRRSV), classical swine fever virus (CSFV), and porcine circovirus 2 (PCV2). All the animal samples were collected according to the protocol approved by the Animal Care and Ethics Committee of National Research Center for Veterinary Medicine (Permit 20170625005).

### Virus and Cells

The PRV variant HN1201 (GenBank accession no. KP722022.1) isolated in 2012 has been described previously (15). Pig kidney cells (PK-15 cells, ATCC® CCL-10) and African green monkey kidney (Vero) cells were grown in Dulbecco's modified Eagle medium (DMEM) (Gibco, CA, USA) supplemented with 10% fetal bovine serum (FBS) (Gibco), and then incubated in a humidified incubator with 5% CO_2_, while the cell culture medium used during viral infection was the DMEM supplemented with 2% FBS.

### Generation of rHN1201^TK–/gE–/gI–/11k–/28k–^

The *Escherichia coli* (*E. coli*) competent cells DY380 that harbor the plasmid pBAC-HN1201^TK−^ were obtained as described previously (15). The DY380 cells were electroporated with a PCR product, a positive selection marker of ampicillin (Amp) gene containing the short extensions (SE) that are homologous to the gI and 28k genes in both ends, to produce intermediate plasmid pBAC-HN1201^TK−/*gE*−/*gI*−/11*k*−/28*k*−/*Amp*+^. The positive clones were selected on agar plates containing 100 μg/ml Amp and 30 μg/ml chloramphenicol, and further confirmed by digestion of *Bam*HI. After digestion using I-SceI, the linear plasmid was transformed into DY380 cells to remove the Amp gene and get the plasmid pBAC-HN1201^TK−/*gE*−/*gI*−/11*k*−/28*k*−^.

To remove the BAC gene cassette and the Loxp residual sequence, a PCR product [a cassette of kanamycin (Kan) gene with homologous arm sequence of Cat-OriS from pBeloBAC11 plus the inverted repeat fragment of the homologous arm of PRV TK gene] was transformed into the DY380 cells containing the plasmid pBAC-HN1201^TK−/*gE*−/*gI*−/11*k*−/28*k*−^ to produce pBAC-HN1201^TK−/*gE*−/*gI*−/11*k*−/28*k*−/*Kan*+^. After digestion using I-SceI, the linear plasmid was transformed into DY380 cells to remove the Kan gene, and finally to produce pBAC-HN1201^TK−/*gE*−/*gI*−/11*k*−/28*k*−^. After that, the positive clone of pBAC-HN1201^TK−/*gE*−/*gI*−/11*k*−/28*k*−^ was transfected into Vero cells to produce markerless 5-gene deleted virus rHN1201^TK−/*gE*−/*gI*−/11*k*−/28*k*−^, which was obtained after 3 rounds of purification by plaque assay.

### *In vitro* Growth Properties and Plaque Morphology

One-step growth curve of the rescued rHN1201^TK−/*gE*−/*gI*−/11*k*−/28*k*−^ was assessed, and then was compared with that of the parental virus HN1201. After infection by the parental and rescued virus (MOI of 1.0), the supernatants of Vero cells were harvested at 0, 4, 8, 12, 16, 20, 24, 28, 32, and 36 h post-infection (hpi) and stored at −80°C. The virus titers were determined by the 50% tissue culture infectious dose (TCID_50_). Growth kinetics for each virus were tested in triplets and the resulting titers were averaged.

Plaque sizes were determined at 48 hpi in Vero cells. Briefly, in the 6-well plates with monolayer cells, the culture medium (DMEM supplied with 2% FBS) containing 1.0 × 10^3^ TCID_50_ of the virus was aspirated at 1 h after incubation, and then the cells were overlaid with 1% low-melting-point agarose in DMEM supplied with 2% FBS for plaque formation. For each virus, 100 plaques were randomly selected, and the plaque size was determined by ImageJ software (National Institutes of Health).

### Animal Experiment

A total of 15 pigs (28-day-old), free of PRV, PRRSV, CSFV, and PCV2, were randomly divided into 3 groups (*n* = 5). The piglets in groups 1 and 2 were vaccinated intramuscularly with 1.0 × 10^5.0^ TCID_50_ rHN1201^TK−/*gE*−/*gI*−/11*k*−/28*k*−^ and one dose of Bartha-K61, respectively. DMEM medium was used as the placebo in group 3 (unvaccinated). After vaccination, rectal temperature and clinical signs were recorded daily. The pig serum samples in days post-vaccination (dpv) of 0, 8, 10, 12, 14, and 21 were collected to monitor gB and neutralizing antibodies (NAbs). All pigs were challenged with HN1201 (1.0 × 10^6.0^ TCID_50_ per pig) intranasally at 21 dpv. At 14 days post-challenge (dpc), all pigs were euthanized and necropsied, and organ samples were collected for immunohistochemistry (IHC) assay.

### Antibody Testing

For antibody testing, gB antibodies of the serum samples were evaluated by the Aujeszky gB (Pseudorabies Virus) Antibody Test Kit (BioChek, The Netherlands) according to the instructions. The PRV-specific NAbs titers were tested by serum-neutralization test (SNT). Briefly, serum samples were inactivated at 56°C for 30 min prior to the SNT. Two-fold serially diluted serum (50 μl) was mixed with an equal volume of the HN1201 or Bartha-K61 (1.0 × 10^2^ TCID_50_) in 96-well plates and incubated at 37°C for 1 h in an atmosphere with 5% CO_2_. After incubation, 100 μl of PK-15 cell suspension containing 2.0 × 10^4.0^ cells was added to each well. The inoculated cells then were incubated at 37°C in an atmosphere with 5% CO_2_ for 5 days, and the titers of PRV-specific NAbs were determined based on cytopathic effect (CPE), and the titers were expressed as the reciprocal of the highest dilution at which infection of the PK-15 cells was inhibited in 50% of the culture wells.

### qPCR

The viral loads in the tissue samples of brain and lung from pigs were tested by qPCR, using the primers specific for gB gene, gB-f (5′-ACAAGTTCAAGGCCCACATCTAC-3′), gB-r (5′-GTCYGTGAAGCGGTTCGTGAT-3′), and Probe-gB (FAM-ACGTCATCGTCACGACC-TARAM), on the CFX96 Touch Real-Time PCR Detection System (Bio-Rad). The copy number for each sample was expressed as log10 copies per gram of samples.

### Immunohistochemistry Assay

Brain, cerebellum, tonsils, and lung samples were collected from the pigs from 3 groups for IHC assay. The samples were fixed with 10% formaldehyde, processed into paraffin blocks, and cut into sections. The sections were stained with hematoxylin and eosin. PRV antigen in the infected pigs' tissues was detected using an IHC with a PRV monoclonal antibody 3B5 (a gB-specific IgG antibody), as described previously (28, 29), and the HRP goat anti-mouse IgG (BTI, USA) served as the secondary antibody.

### Statistical Analysis

Data were presented as mean ± SD. The survival rates were analyzed by the Kaplan–Meier test. The differences in plaque areas of viruses, body temperature, and antibody titers of piglets between groups were determined by using Student's *t*-test. Differences were considered statistically significant when *p* < 0.05.

## Results

### Rescue of rHN1201^TK–/gE–/gI–/11k–/28k–^ and Growth Properties

**Figure 1:**
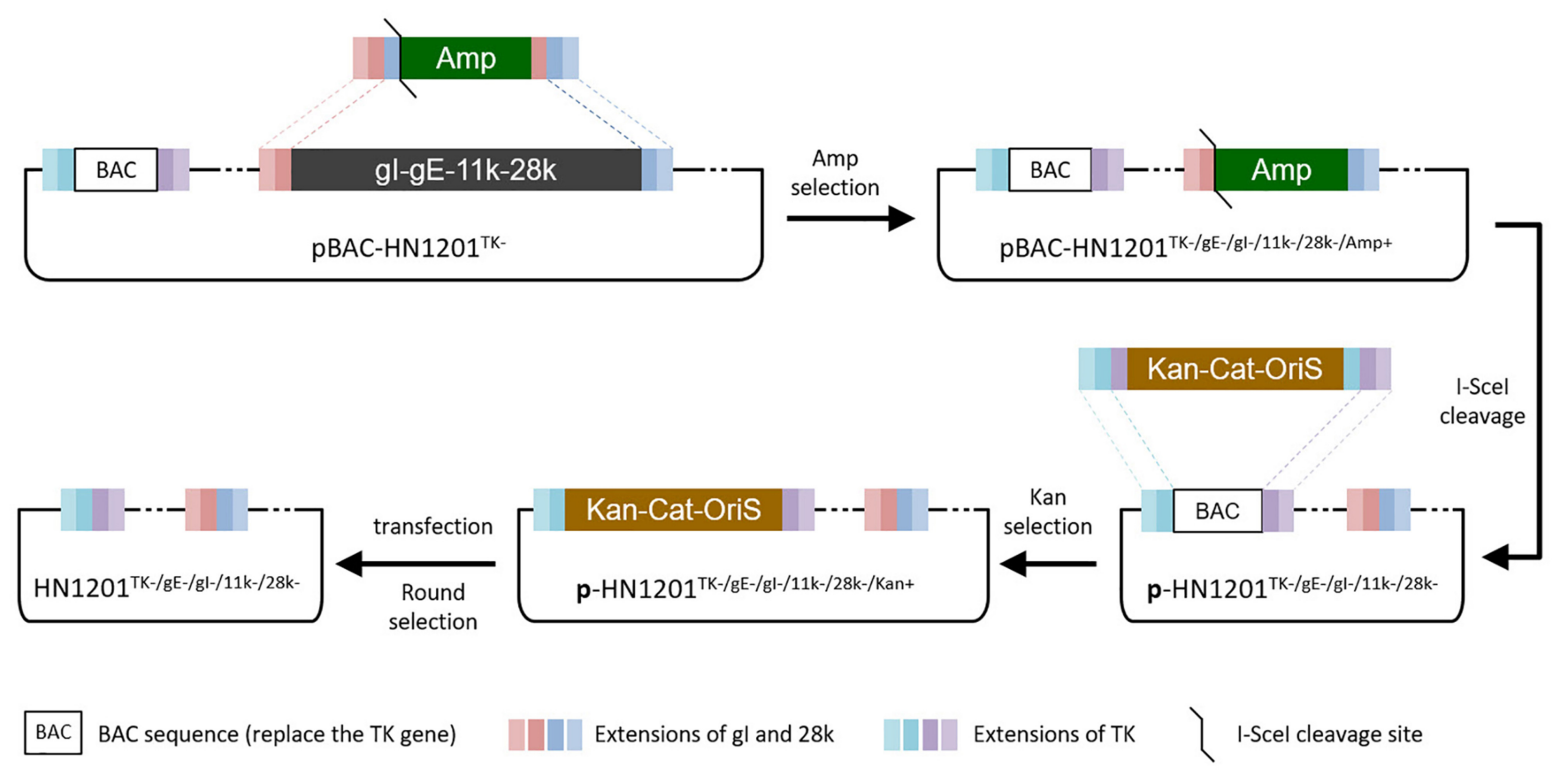
Overview of strategies for the TK/gE/gI/11k/28k deletion. Boxes of the same color represent identical sequences.

**Figure 2:**
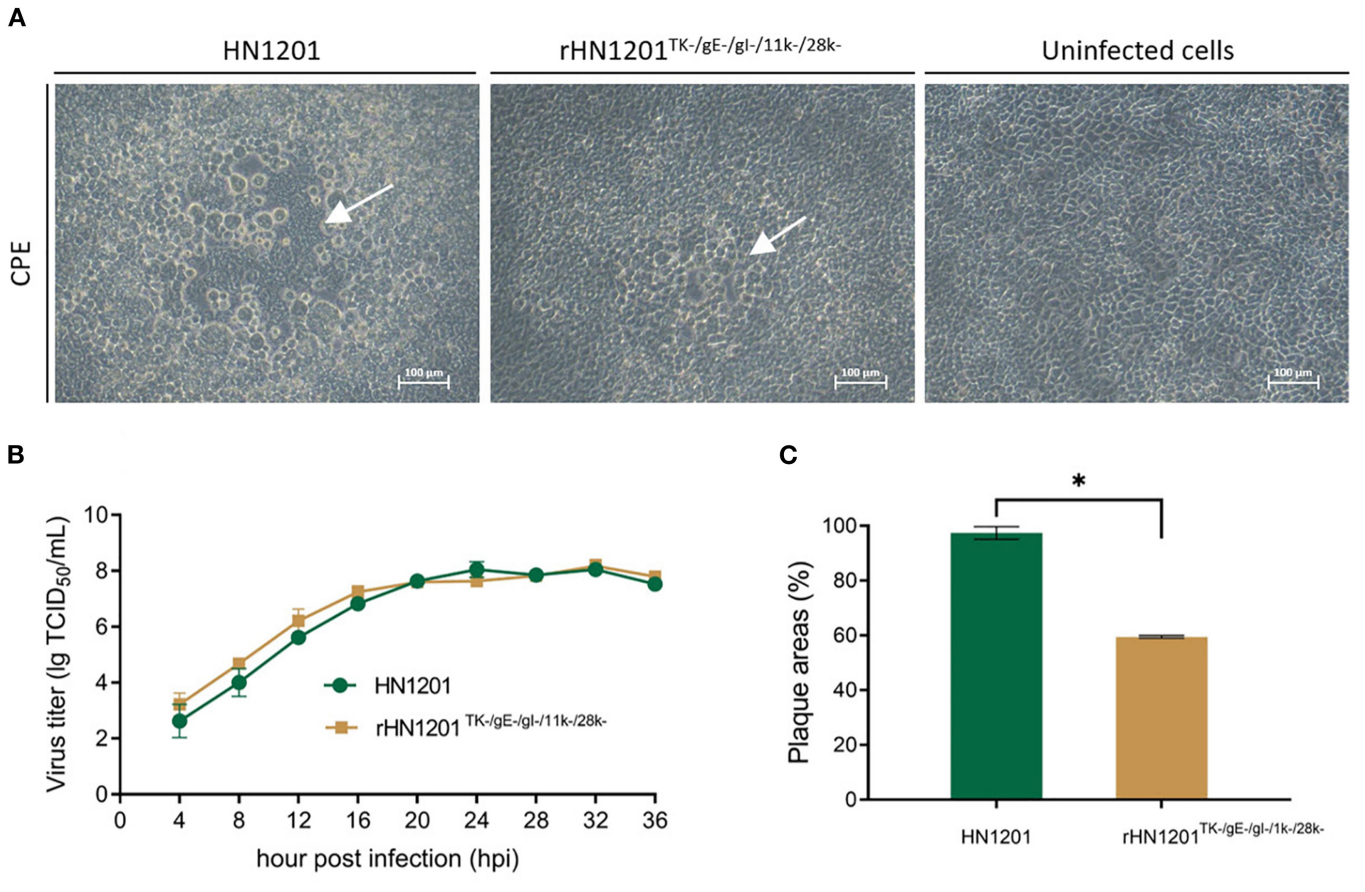
Rescue and the characterization of recombinant PRV. **(A)** Cytopathic effect in Vero cells after transfection with recombinant PRV pBAC plasmids. The white arrow indicated the CPE. Bar = 100 μm. **(B)** Multiple growth curves of the chimeric viruses. The culture supernatants were collected at the indicated time points for the viral titer determination. **(C)** Plaque size of the recombinant viruses. The plaques were measured at 48 hpi. The plaque size induced by the parental virus was set at 100%. Asterisk denotes a statistically significant difference (*p* < 0.05).

The PCR product of SE(gI)/I-SceI/Amp/SE(28k) was applied to replace the fragment SE(gI)/gI/gE/11k/28k/SE(28k) in pBAC-HN1201^TK−^ by homologous recombination in *E.coli* DY380 (Figure 1). The 5-gene deleted plasmid pBAC-HN1201^TK−/*gE*−/*gI*−/11*k*−/28*k*−/*Amp*+^ was selected on an agar plate containing chloramphenicol and ampicillin, and further confirmed by sequencing. After digestion using I-SceI, the linear plasmid was transformed into *E. coli* DY380 to remove the Amp gene, and then the plasmid pBAC-HN1201^TK−/*gE*−/*gI*−/11*k*−/28*k*−^ was generated. The CPE could be observed at 72 hpi (Figure 2A). Plaques of rescued PRV were isolated and subjected to three rounds of purification. The virus was named rHN1201^TK−/*gE*−/*gI*−/11*k*−/28*k*−^, which lacked TK, gE, gI, 11k, and part of the 28k genes compared with the parental virus HN1201.

As shown in Figure 2B, the growth features of rescued virus rHN1201^TK−/*gE*−/*gI*−/11*k*−/28*k*−^ were virtually identical to that of parental virus HN1201 in PK-15 cells. However, the plaque areas of the rescued virus were smaller than those formed by HN1201 in PK-15 cells (Figure 2C).

### Protection of Vaccinated Pigs After Challenge

**Figure 3:**
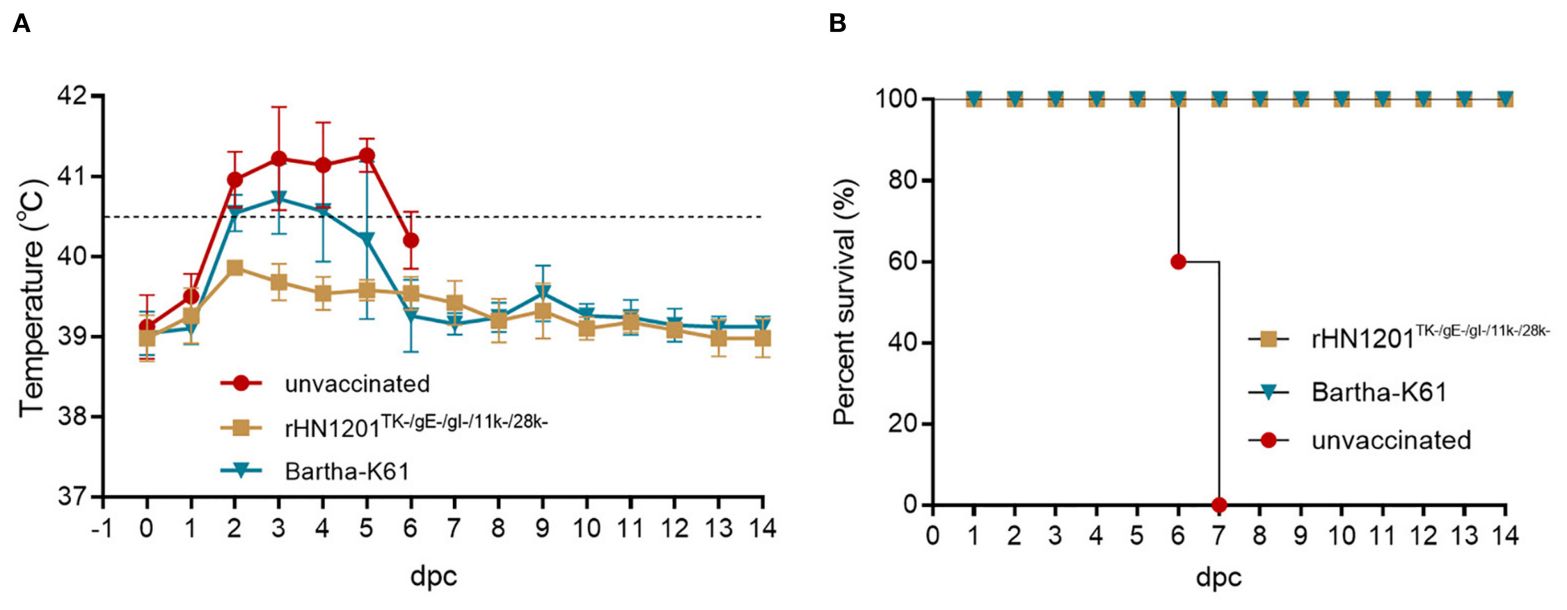
Body temperature and survival rate of pigs after challenge. **(A)** Body temperature of pigs challenged with HN1201. **(B)** Survival rate of pigs challenged with HN1201. The survival rates were analyzed by the Kaplan Meier test.

To determine the immunogenicity of rHN1201^TK−/*gE*−/*gI*−/11*k*−/28*k*−^, 15 piglets were selected for vaccination with rHN1201^TK−/*gE*−/*gI*−/11*k*−/28*k*−^ and Bartha-K61, and subsequent challenge with the HN1201 strain at 21 dpv. The results showed that no clinical symptoms were observed in all pigs after vaccination. After challenge with HN1201 intranasally, all pigs in group 3 (unvaccinated) exhibited high fever (40.5–41.7°C, Figure 3A), depression, anorexia, cough, and systematic neurological signs like convulsion and ataxia. All the unvaccinated pigs died at 6–7 dpc, and no pigs died after being vaccinated with rHN1201^TK−/*gE*−/*gI*−/11*k*−/28*k*−^ or Bartha-K61 (Figure 3B). As with the clinical signs of group 3 at 2–6 dpc, the pigs in group 2 (Bartha-K61) showed a transient period of high fever after challenge (three out of five pigs showed the temperature higher than 40.5°C for 4 days), and all pigs recovered from 7 dpi. In contrast, pigs in group 1 showed no clinical signs throughout the whole experiment. The result indicates that the vaccination with rHN1201^TK−/*gE*−/*gI*−/11*k*−/28*k*−^ could protect pigs against challenges with the new virulent PRV strain.

### Antibody Response After Vaccination and Challenge

**Figure 4:**
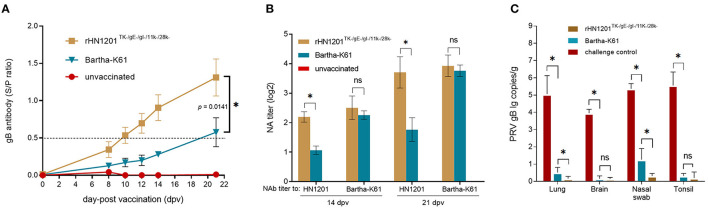
Production of PRV-specific antibodies after vaccination and viral load test of pigs after challenge. **(A)** The gB antibodies were detected at the indicated time points after vaccination. The ELISA values of pig serum samples are given as S/P ratios, S/P > 0.5 was considered positive. **(B)** NAbs against the HN1201 and Bartha-K61 in the indicated groups at 21 dpv and 35 dpv (14 dpc). Standard deviations are shown as error bars. The *t*-test was performed for statistical analysis. **(C)** Viral load detection of pigs after challenge with HN1201. The viral loads between different groups were analyzed by *t*-test. Asterisk denotes a statistically significant difference (*P* < 0.05), ns indicated no significant difference.

After vaccination, the levels of gB antibody increased in all the vaccinated groups. The results showed that the gB antibodies elicited by rHN1201^TK−/*gE*−/*gI*−/11*k*−/28*k*−^ in the serum samples were all positive at 10 dpv and with the highest level at 21 dpv before challenge (*S*/*P*-value of 1.4), which were earlier and higher than that of the Bartha-K61 group (not fully positive and *S*/*P*-value of 0.6 at 21 dpv) (Figure 4A), indicating an enhanced protective effect of the recombinant PRV strain. The anti-PRV NAbs of pig serum elicited by rHN1201^TK−/*gE*−/*gI*−/11*k*−/28*k*−^ vaccination showed a high level against both the HN1201 and Bartha-K61 strains at 14 and 21 dpv, while anti-PRV NAbs of pig serum from the Bartha-K61 vaccination group showed a significantly lower NA titer against HN1201 (Figure 4B), which indicated an insufficient effect of Bartha-K61 for protecting animals from infection of epidemic strains. Of note, no gB-specific antibodies and NAbs were detected in the unvaccinated group.

### Viral Load Assay After Challenge

After the challenge, the viral loads of the tissue samples were detected in the DNA levels by qPCR. The results showed a significantly higher viral load in the lungs and nasal swab of the piglets in the rHN1201^TK−/*gE*−/*gI*−/11*k*−/28*k*−^ group than those in the Bartha-K61 group (*p* < 0.05) (Figure 4C), but not in the tissues of the brain and tonsil, which might be caused by the easier exposure of the respiratory tract to PRV.

### The Result of IHC Assay After Challenge

**Figure 5:**
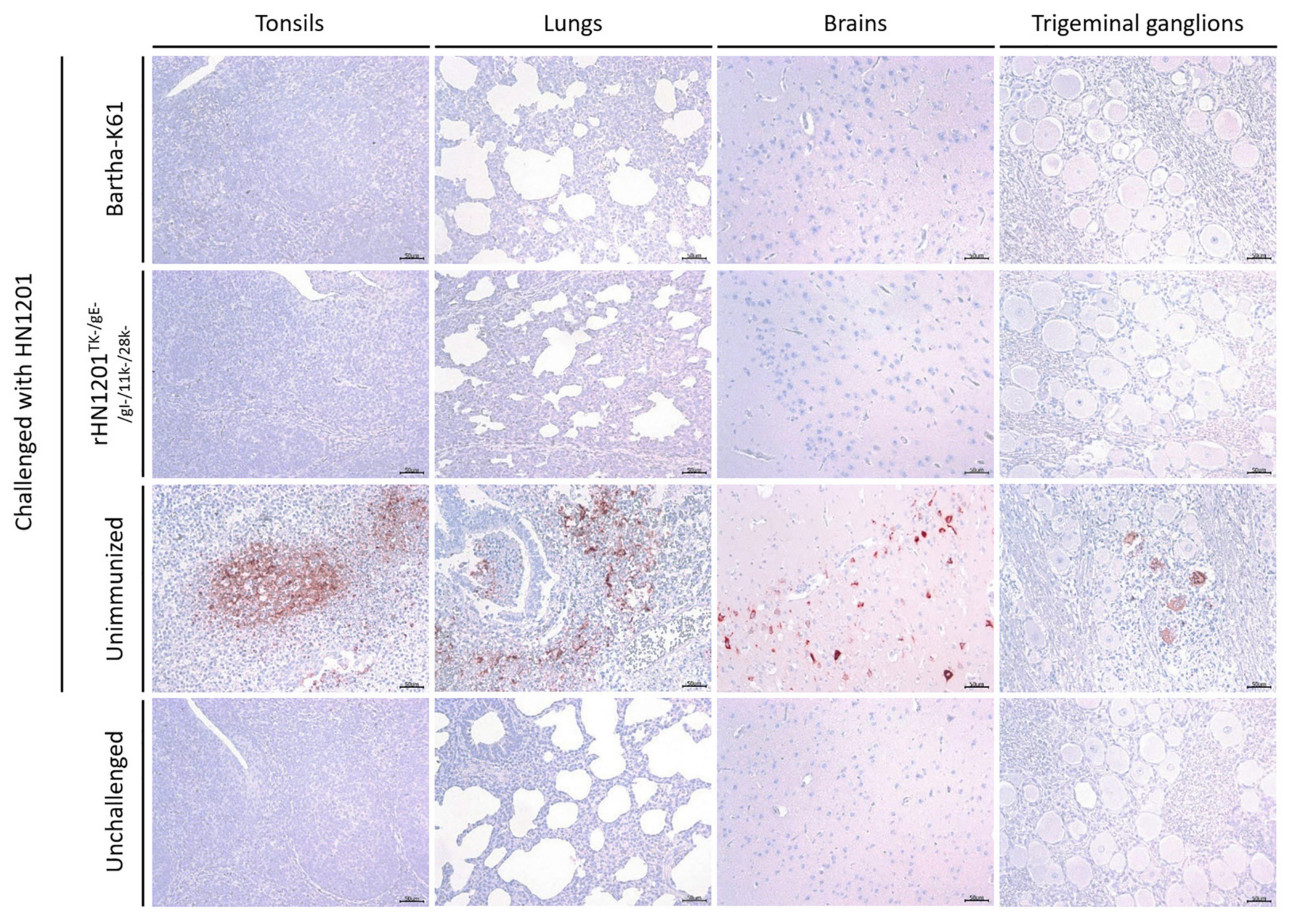
Results of IHC assay of tonsils, lungs, brains, and trigeminal ganglions. Representative IHC images of tonsils, lungs, brains, and trigeminal ganglions were shown corresponding to animal groups infected with HN1201. The groups' names were indicated on the left of the figure. Bar = 50 μm.

In the IHC assay of tissue samples, as shown in Figure 5, the pigs in the unvaccinated group showed strong positive reaction in the tonsil, lung, brain, and trigeminal ganglion. While no positive reactions was detected in the tissues of unchallenged and vaccinated pigs recovered after the challenge, which indicated a sufficient protection effect of vaccination of rHN1201^TK−/*gE*−/*gI*−/11*k*−/28*k*−^.

## Discussion

The widely used PRV vaccines of classical strain, such as the strain Bartha-K61, were effective in PR control during the past several decades in China (7, 8). However, the re-emergent outbreaks of PR in the Bartha-K61 vaccinated pig farms since late 2011 indicated the insufficient protection of classical PRV vaccines (10–12, 16). In this study, a TK/gE/gI/11k/28k deleted PRV strain, rHN1201^TK−/*gE*−/*gI*−/11*k*−/28*k*−^, was generated based on the RPV variant strain HN1201. The full protection in pigs immunized with rHN1201^TK−/*gE*−/*gI*−/11*k*−/28*k*−^ indicated that it is a safe and protective vaccine candidate to control the PR caused by new epidemic PRV variants.

The 3-kb deletion in the unique short (US) region of Bartha genome, partial loss of 28k, most of US7 (gI), and complete deletion of US8 (gE) and 11k (9) indicated an important role of these genes in the virulence determinant during infection. So, the genes of gI, gE, 11k, and 28k were selected for the construction of the 5-gene deleted PRV. The absence of the glycoprotein gE/gI complex in the Bartha genome partly explains the increased type I interferon response by plasmacytoid dendritic cells, and the potential of PRV Bartha vaccine strain to induce a strong type I interferon may contribute to the efficacy of the highly successful vaccine (9). In the future, the immunogenicity of the PRV 5-gene deleted vaccine candidate will be evaluated at the level of cellular immunity.

As reported, PRV gE and gI are required for efficient cell-to-cell spreading, gE/gI participates in the envelopment of nucleocapsids into cytoplasmic membrane vesicles (30), and delivery of virus particles to cell junctions would enhance virus spread (31). The viral protein of 11k interacts with a microtubule motor Kif1a to mediate virus transport (32); this mechanism could be strengthened by other viral proteins such as gE and gI (33). The interaction among these viral proteins reveals the transmission mechanism of PRV and provides a perspective to understand PRV virulence.

In herpes simplex virus, gI and gE null mutants lead to the formation of small plaques (34), and in PRV, the deletion of gI/gE/TK/UL13 also resulted in the formation of small plaques (35), which was consistent with the findings in our study. An attenuated PRV strain with the deletion of US8/11k/28k genes results in a higher titer and larger plaque size than that of WT treatment in Vero cells (36), while the larger size of the plaque formed by JS-2012 was considered induced by the cell adaption in Vero cells (120 passages).

The rapid improvement of biotechnology promoted the research of genetically modified PRV in recent years, such as the application of BAC system in gene deletion (16, 37–39), and the identification of foreign gene insertion sites in a PRV vector (40) and non-coding regions (UL11-10, UL35-36, UL46-27, or US2-1). Notably, the method that allowed PRV genome manipulation by using the CRISPR/Cas9 system in PK15 cells was developed (23, 41) and showed a high positive rate without constructing homology arms, offering a simple and efficient method to manipulate the viral genome in the future, especially in the identification of potential new virulence genes for the highly safe vaccine development to control PR.

In summary, here, a recombinant PRV rHN1201^TK−/*gE*−/*gI*−/11*k*−/28*k*−^ was constructed by the BAC system, which elicited an earlier and higher level of gB antibody, and the NAbs elicited by rHN1201^TK−/*gE*−/*gI*−/11*k*−/28*k*−^ were effective against both PRV classical and variant strains. The rHN1201^TK−/*gE*−/*gI*−/11*k*−/28*k*−^ vaccination could provide effective protection against the challenge with the PRV variant. Therefore, it is a promising vaccine candidate for the prevention of the current epidemic of PR in China.

## Data Availability Statement

The original contributions presented in the study are included in the article/supplementary material, further inquiries can be directed to the corresponding authors.

## Ethics Statement

The animal study was reviewed and approved by Animal Care and Ethics Committee of National Research Center for Veterinary Medicine.

## Author Contributions

FT, BH, and KT conceived and designed the research. SY, XB, YZ, LG, TW, and YW conducted the experiments. FT, YZ, and BH analyzed the data. SY, FT, BH, and KT conceived the study, carried out additional analyses, and finalized the manuscript. All authors contributed to the article and approved the submitted version.

## Funding

This study was supported by the project of R&D and industrialization of genetically engineered vaccines for swine pseudorabies, swine ring, and *Mycoplasma hyopneumoniae* (201200211200).

## Conflict of Interest

The authors declare that the research was conducted in the absence of any commercial or financial relationships that could be construed as a potential conflict of interest.

## Publisher's Note

All claims expressed in this article are solely those of the authors and do not necessarily represent those of their affiliated organizations, or those of the publisher, the editors and the reviewers. Any product that may be evaluated in this article, or claim that may be made by its manufacturer, is not guaranteed or endorsed by the publisher.
